# Case Bronchitis—Pleurisy, with Effusion—Pneumothorax—Death

**Published:** 1856-10

**Authors:** Ralph N. Isham

**Affiliations:** Chicago, Ill.


ARTICLE VI.—*Case Bronchitis—Pleurisy, with Effusion—Pneumothorax—Death.* By RALPH N. ISHAM, M. D., Chicago, Ill.

James Marr, aged 40, married, a native of Ireland, by occupation a stone-dresser, was first seen by me Jan. 15th, 1856.

No hereditary tendency to disease traceable; both of his parents died at an advanced age of life.

The patient, a muscular, well-developed man, of medium stature, states that "he has never been ill a day in his life," until his present attack. Is not a temperate man, and at times indulges *freely* in intoxicating drinks.

Ten days previous to my first visit, (Jan. 5th,) after exposure to wet, he was siezed with "shiverings of cold," lasting some hours, and followed by a sharp pain in the left side, aggravated by deep inspirations. He states that he was bled freely from both arms, and blistered by his medical attendant, who, for some unknown reason, abandoned his case.

*Present Condition.*—He complains of pain in the left side, a severe and harassing cough, accompanied with a viscid, frothy, white expectoration, showing no blood under the microscope, and which has been present from the commencement of the disease; skin hot and dry; pulse full, soft, and accelerated; tongue coated with a greasy white fur, edges red; bowels regular; appetite poor; insomnia.

*Physical Signs.*—Left side, posteriorly, from inferior angle of scapula to the base of the lung, dulness on percussion; over a space, bounded by the dulness superiorly and inferiorly, and extending three inches from the spine, is heard bronchial respiration, but none heard over the left auxiliary and lateral regions; increased vocal resonance over space above bounded. Sibilant and sonorous rales heard over the whole chest. Ordered C.C., (dry,) xij., to each chest, and ipecac., with Dover's powder every third hour.

*Jan.* 17.—Pleuritic effusion in left chest has taken place; ordered diuretics and flying blisters. Feels much improved, pain in the chest absent, and considerable moisture of surface.

19*th.*—Has grown gradually weaker; fluid increased; considerable dyspnœa; profuse perspiration; tongue still coated, and no appetite. Moderate stimulus added to treatment, with generous diet.

23*d*.—Dyspnœa increasing. Œdema of feet and ancles first noticed; no œdema of chest. Tongue coated, pulse rapid and feeble. The fluid has reached the middle of dorsum of scapula. The heart's apex beats at junction of fifth rib and sternum—left side. Bronchial respiration is heard over a space one inch square, at level of fluid, and about four inches from the spine. Sonorent and sibilant rales diminished, but not entirely absent.

23*d*, *Vespere*.—Patient suffers from orthopnœa; the countenance is anxious and of a dusky hue; pain on percussion of left chest, amphoric respiration, and metallic voice, and cough heard on left side. No metallic tinkling; no resonance on percussion; no change in the expectoration.

Considerably relieved by the use of proper remedies.

28*th*.—Physical signs the same, with the heart further displaced; apex pushed to the right side, having, as it were, swung upon its axis. Tympanitic percussion in front of left chest.

The patient had refused to submit to the operation of thoracentesis, being somewhat relieved by the use of proper remedies; but, on the 7th of February, he became suddenly worse, and died.

Duration of illness, 33 days.

*Sectio Cadaverous*—20 *hours Post Mortem*.—A trocar introduced into the left chest, between the eighth and ninth ribs, posteriorly, (erect posture,) gave exit to pus, in amount 40 ℥. The lung was found pushed up to the anterior superior portion of chest, adherent to the front and side, but not behind. A small strip of lung extended posteriorly nearly to the diaphragm, and was adherent to that muscle by a dense band of false membrane. The remainder of the chest was filled with pus and air.

The blowpipe, introduced into the trachea, inflated both lungs tolerably well, but failed to detect the point of perforation. The trachea was then dissected up, and the blow-pipe introduced into the left bronchus, when bubbles of air arose through the water placed over the lung, showing the perforation which had taken place at the point where the bronchial respiration had been so persistent during life. The opening was valvular, and concealed by false membrane. No tubercles existed in either lung. Heart natural; liver large and fatty; kidneys slightly granular, and weighing 8¼ oz. each.

The occurrence of bronchial respiration in pleuritic effusion, is not a rare phenomenon, and, as is illustrated by this history, may occur in *recent* as well as in chronic cases. In two examples recorded by Dr. Graves, the usual signs of effusion were well marked, and, being of an urgent nature, paracentesis would have been performed but for the presence of this bronchial respiration, which was heard over the anterior portion of chest in a line drawn vertically through the mammary region, and also posteriorly above and below the scapula. On dissection of these cases, which was similar in both, "a strong adhesion extended from two inches below the clavicle of the affected side in a line passing through the middle of the mammary region, nearly to the bottom of the anterior part of the lung." "This adhesion, about two inches in breadth, was very firm and close, so as to form an intimate union between the pulmonary substance and the anterior parieties of the chest, and extended nearly from the apex of the lung to its base. Along this line the pulmonary tissue formed a plate of compressed lung, two inches in thickness, which, like a vertical partition, divided the pleural cavity into two chambers, each filled with sero-purulent matter, and separated by the lungs, extending from its root to its anterior adhesions."*

Its presence in these cases, as well as the one under consideration, may, perhaps, be partially accounted for by the entrance of air into the larger bronchial tubes, which were not obliterated; but, why it is so often absent, is still unexplained, and can only be solved by careful observation.

The absence of rales, the displacement of the heart, and the bulging of the intercostal spaces, are the signs upon which we are chiefly to rely in distinguishing the bronchial respiration of pleurisy from that of pneumonia. In the resolution of the lat-

* Dublin Hospital Reports, Vol. V.

ter, its disappearance is accompanied by the rales crepitans redux, which are of course absent in pleurisy.

This case, furthermore, shows, that the obliteration of the intercostal spaces is not necessarily present in cases of empyema even when the heart is displaced and the lung much compressed. And from the fact that it does not occur in pneumonia; that in emphysema it is accompanied with resonance on percussion; and that in simple pneumothorax, although the action of air upon the pleura gives rise to rapid effusion, supplanting the resonance by dulness, it may be distinguished by Hippocratic succussion whether the perforation is closed or not, renders this a valuable sign where present. Its absence in this case tends to establish the correctness of Dr. Stokes' opinion, that it is caused by paralysis of the intercostal muscles, produced by inflammation of the pleura; for effusion to a great extent was undoubtedly present, accompanied with eccentric pressure, necessarily great on account of the valvular arrangement of the perforation, which, whilst it readily admitted the air in the chest, prevented its easy egress.

On reviewing this case, no question as to the perfect propriety of paracentesis can be raised, had the patient consented, occurring, as it did, in a good subject, without tuberculosis, and a fluid of a purulent character.

